# Complex Networks in Contemporary Science and Technology

**DOI:** 10.3390/e28040456

**Published:** 2026-04-16

**Authors:** L. H. A. Monteiro

**Affiliations:** 1 Escola de Engenharia, Universidade Presbiteriana Mackenzie, São Paulo 01302-907, SP, Brazil; luizm@mackenzie.br or luizm@usp.br; 2 Escola Politécnica, Universidade de São Paulo, São Paulo 05508-010, SP, Brazil

Many systems in nature, society, and technology can be represented as networks of interacting components. Over the past two decades, complexity science has provided a conceptual framework for understanding how interactions among many elements generate collective behavior [1,2,3,4]. Within this perspective, network representations offer a suitable way to investigate how connectivity patterns shape the dynamics and organization of large interconnected systems. They also help reveal how the dynamics of individual elements may modify local connectivity and, consequently, the structure of the network. Theoretical models and analytical studies play an important role in this context, helping to uncover the mechanisms that govern the emergence of collective phenomena.

Several contemporary problems can be viewed through the lens of complexity science and complex networks. Some of these are listed below:

1. **Stability of power grids with heterogeneous energy sources.** Modern power grids operate with a heterogeneous portfolio of generation technologies, including wind, solar, nuclear, hydroelectric, and coal plants [5]. These sources exhibit different dynamical characteristics, ranging from intermittent renewable generation to conventional synchronous generators that provide inertial support and frequency regulation. In addition, the transition toward smart grids, which integrate distributed generation, advanced sensing, and digital control, is further increasing the complexity of power system operation [6]. Electricity demand is also rising, driven by the expansion of electric vehicles, large-scale data centers, and artificial intelligence systems [7,8]. This combination of heterogeneous generation, distributed resources, and time-varying loads poses significant challenges for grid stability. It requires new approaches to the modeling, control, and real-time coordination of large interconnected power networks, where intermittent generation and reduced system inertia may lead to loss of synchronization and cascading failures.
2. **Distributed control of large-scale communication systems.** Modern communication and control infrastructures operate in environments where multiple technologies coexist [9]. Current networks integrate legacy systems such as 4G with newer standards like 5G and the emerging 6G architecture, alongside Wi-Fi, large constellations of thousands of satellites, and billions of mobile devices [10,11]. Moreover, the expansion of the Internet of Things and the increasing adoption of autonomous vehicles, robotic systems, and drones introduce massive numbers of sensors and agents that exchange information and make decisions in real time [12]. Managing such heterogeneous and densely interconnected systems requires distributed control strategies capable of coordinating many interacting components. These strategies must cope with communication delays, interference, and variable connectivity, and must also ensure reliability, efficiency, and scalability.
3. **Dynamics of ecosystems.** A central question in ecology is the stability and resilience of ecosystems in the presence of environmental disturbances. Ecosystems are composed of many species whose interactions form complex networks of predation, competition, and mutualism. In this context, biodiversity plays a key role: greater species diversity can increase the robustness of ecological systems by providing functional redundancy and alternative pathways for energy and nutrient flows [13,14]. However, ongoing climate change is altering temperature regimes, precipitation patterns, and seasonal cycles, which modifies species distributions and interaction strengths [15]. These environmental shifts may destabilize ecosystems and push them toward critical transitions. In particular, the loss of certain species can trigger cascading extinctions, where the disappearance of one species propagates through the ecological network, leading to further losses [16]. Understanding how biodiversity, climate variability, and network structure interact to determine ecosystem stability and resilience continues to be a major scientific challenge.
4. **Gene and protein interaction networks.** Many cellular processes emerge from networks of interacting genes and proteins instead of the action of isolated molecules. Genes interact through regulatory networks, while proteins form extensive interaction and signaling networks that control cellular functions [17]. Open questions include how the structure and dynamics of these networks determine cellular stability and how genetic mutations or environmental perturbations propagate through them, potentially leading to diseases such as cancer or neurodegenerative disorders [18,19]. Additionally, advances in experimental techniques and the growing body of genetic knowledge are driving the design of increasingly complex artificial gene regulatory networks [20]. However, interpreting the large-scale datasets produced by modern genomics and proteomics and identifying the key interactions that govern cellular behavior remain important problems at the intersection of complexity science, biology, and medicine.
5. **Biological and artificial neural networks.** The brain is a paradigmatic complex system composed of vast networks of neurons whose collective activity underlies perception, cognition, and behavior [21]. These biological neural networks exhibit intricate connectivity patterns, nonlinear dynamics, and adaptive plasticity, allowing information to be processed and integrated across multiple spatial and temporal scales [22]. Central questions concern how coordinated neural activity emerges from the interactions of many simple units and how such an activity supports learning, memory, emotion, and decision-making. Progress in neuroscience and machine learning has also enabled the development of increasingly sophisticated artificial neural networks capable of performing tasks such as image recognition, natural language processing, and strategic planning [23]. Current challenges involve understanding the principles that link the organization of biological neural networks to artificial architectures and investigating whether artificial networks could eventually exhibit forms of autonomous reasoning or conscious awareness [24].
6. **Propagation of diseases and information on complex networks.** The spread of infectious diseases and information is strongly influenced by the complex networks that connect individuals through social contacts, transportation systems, and digital communication platforms. Modern societies are characterized by highly heterogeneous and dynamic connectivity patterns, with billions of people interacting through social media, air travel, and mobile communication, creating multiple pathways for rapid propagation. A central question is how network structure, mobility patterns, and behavioral responses affect the speed and scale of spreading processes [25,26,27,28]. These issues encompass predicting epidemic outbreaks in globally connected populations, understanding the impact of misinformation and rumor transmission in online networks, and designing effective intervention strategies (such as vaccination policies, targeted isolation, or content moderation) capable of limiting large-scale propagation in such systems.
7. **Complexity in socioeconomic systems.** Social and economic systems arise from the behavior of large numbers of individuals linked through institutional, financial, and communication structures. From a complexity science perspective, collective dynamics such as opinion formation, cooperation, market fluctuations, and technological adoption result from decentralized interactions rather than centralized control. A key issue concerns how local decision-making processes give rise to large-scale social and economic patterns. Important challenges include the emergence of political polarization and echo chambers in online platforms, the dynamics of collective behavior in digital environments, and the formation of speculative bubbles and market instabilities [29,30,31]. Another emerging phenomenon is the rise of cryptocurrencies, which create decentralized financial systems where the value, stability, and adoption of digital assets depend on complex interactions among users, miners, markets, and information exchange [32]. Explaining how network organization, incentives, and information flows drive these processes has become a major line of research in the socioeconomic sciences.

These topics illustrate how network structure and interaction dynamics shape the collective behavior of complex systems in our world. Two other topics are the evolution of urban road infrastructure [33] and the robustness of supply and logistics chains [34].

The articles in the Special Issue *Dynamics in Biological and Social Networks* illustrate the broad applicability of complex-systems approaches and information-theoretic methods across biological, social, and technological domains. One study in the Special Issue investigates an evolutionary spatial version of the Ultimatum Game in which agents endowed with basic emotions (such as anger, fear, joy, sadness, and surprise) interact on a lattice, exploring how emotional strategies influence cooperation, wealth distribution, and the emergence of fairness [35]. Another study models the spread of infectious diseases in urban populations by combining complex network models with genetic algorithms to estimate network parameters that reproduce realistic epidemic dynamics [36]. Opinion formation in social systems is examined through a model incorporating three-stage cascade information attenuation and opinion leaders, illustrating how trust, leadership influence, and network connectivity affect the convergence and polarization of opinions [37]. A further study explores the intersection between mathematical epidemiology and chemical reaction network theory, arguing that epidemiological models can benefit from the formal language and analytical tools of reaction networks, particularly for studying stability, reproduction numbers, and essentially non-negative dynamical systems [38]. The Special Issue also includes a study that integrates fuzzy belief structures with the TOPSIS decision-making method to analyze airline rankings from large-scale online reviews, revealing how entropy can capture the diversity and consistency of customer satisfaction in digital feedback systems [39]. Another contribution examines eye fixation durations during visual exploration using a competition model between neural networks responsible for fixation and saccadic movements, showing that this competition can reproduce empirical distributions of fixation times [40]. The final contribution analyzes the structure of online communication networks by introducing the Structural Entropy Index of a Community (SEIC), an entropy-based metric that quantifies the decentralization of communication within social groups and helps identify influential actors and vulnerability to misinformation [41]. Collectively, these seven articles show how a range of approaches, including complex networks, agent-based models, entropy measures, and computational optimization, provide deeper insights into the dynamics of cooperation, epidemic spreading, opinion formation, information diffusion, collective decision-making, and neural processes in complex adaptive systems.

## Data Availability

Not applicable.
